# The factors influencing the psychological distress of transgender women in Shandong, China: a cross-sectional study

**DOI:** 10.1186/s12889-022-13357-9

**Published:** 2022-05-12

**Authors:** Meng Sun, Haoqiang Ji, Xu Chen, Jia Xu, Jiachen Lu, Yaohui Yi, Yuanping Pan, Ruiheng Wu, Yunting Chen, Yuxin Duan, Xiaofeng Dou, Ling Zhou

**Affiliations:** School of Public Health, Dalian Medical University, Dalian, 116044 Liaoning China

**Keywords:** Transgender women, GHQ-12, Psychological distress, Associated factors, China

## Abstract

**Background:**

As a group at high-risk for acquired immune deficiency syndrome (AIDS) infection, the psychological distress of transgender women cannot be ignored while preventing and controlling AIDS risks. Transgender women are a vulnerable group, and their psychological distress deserves attention. The purpose of this study was to evaluate the psychological distress of transgender women and further determine the influences of factors on the psychological distress of transgender women.

**Methods:**

From March 2021 to August 2021, a cross-sectional survey was conducted in Shandong province, China. Data were collected by a questionnaire designed for transgender women, and the GHQ-12 scale was used to measure their psychological distress. The questionnaire combined sociodemographic characteristics, HIV/AIDS cognition, related behaviors, substance abuse, social support, gender identity and other factors. Univariate logistic regression and multivariate logistic regression models were used to explore the psychological factors of transgender women.

**Results:**

In this study, the rate of transgender women with psychological distress was 20.08%. Earned monthly income between 10,000–15,000 yuan (*OR*:0.16, 95% CI:0.06–0.45) and a monthly income greater than 15,000 yuan (*OR*:0.07, 95% CI:0.01–0.43) were protective factors in the psychology of transgender women. Transgender women who never disclosed sexual orientation and identity (*OR*: 0.19, 95% CI: 0.06–0.58), who only disclosed their sexual orientation and identity to families or friends (*OR*: 0.41, 95% CI: 0.18–0.93) were also less likely to have psychological distress. Additionally, transgender women who did not desire to be identified with their sexual orientation and identity (*OR*: 3.31, 95%CI: 1.08–10.16) and who reported that the Internet did not play an essential role in helping determine sexual orientation (*OR*: 5.96, 95% CI: 2.91–12.20) were more likely to have psychological distress.

**Conclusion:**

Transgender women were at risk of psychological distress. Earning more money can help transgender women's psychological health. When formulating measures for transgender women, we should pay attention to enhance social inclusion and social acceptance of their gender identity and sexual orientation. Strengthening the role of the internet in transgender women's confirmation of sexual orientation and improving the social acceptance of transgender women will have a positive impact on the psychological status of transgender women.

**Supplementary Information:**

The online version contains supplementary material available at 10.1186/s12889-022-13357-9.

## Background

Transgender is a definition of how a person feels about their gender identity [1]. A person is cisgender when their gender at birth matches their assigned gender and transgender when their gender at birth does not match their assigned gender [1]. Transgender women are individuals who were assigned male at birth but identify as female [2, 3]. In addition to psychological gender identity, transgender people may undergo sex-change surgery, take hormones (contraceptives, etc.), and inject their bodies with fillers to change their characteristics to make their bodies conform to their gender identity. Because of the diversity of the community of transgender women, they may not change their appearance and live instead in male-presenting bodies [1].

Transgender women are one of the groups with the highest infection rate of human immunodeficiency virus (HIV) in the world, which has characteristics of concealment and a low detection rate. In a global study of the burden of AIDS (acquired immune deficiency syndrome), the odds of HIV infection among transgender women was 48.8 times that of cisgender adults of reproductive age [4]. Additionally, a meta-analysis showed that the HIV-positive rate among transgender women in 15 countries was 19.1%, the HIV prevalence rate among transgender women in low- and middle-income countries was 17.7%, and the HIV prevalence rate in high-income countries was 21.6% [4]. Transgender women exhibited more high-risk behaviors, such as drug abuse, multiple sexual partners, unprotected sex, etc. [5]. The prevalence rate of HIV among transgender women was significantly higher than that among men who have sex with men (MSM). According to the gender minority stress model of Mayer and others [6], transgender women face greater stress, are more vulnerable to high levels of discrimination and violence, and face a higher risk of HIV infection and transmission [7].In addition, because the gender division is still dualistic, transgender people are excluded from most social infrastructures [8]. A study in the United States reported that transgender people who self-reported falling outside the binary category had worse health outcomes, including mental health, than their binary peers [9]. This increases the risks faced by minority groups, such as transgender people, who already face disadvantages such as discrimination and violence [10]. Research on transgender people showed that they were more likely than cisgender people to be diagnosed with depression and substance use disorders, engage in self-harm and die by suicide [10, 11]. Transgender individuals have worse mental health than cisgender individuals [9, 12]. However, there is little research on transgender women in China, especially on the factors that influence psychology. A study has pointed out that when seeking social support, individuals will choose to make changes to more positive behaviors and find an alternative way to obtain happiness and make a promotional response [13]. There was a moderate correlation between social support and physical and mental health [14]. As a marginalized group, transgender women are vulnerable to discrimination and violence from family and society [15, 16]. At the same time, they are also influenced by their own psychological gender identity and self-doubt, which leads to psychological problems such as anxiety, depression and even suicide.

In addition, because of many differences from traditional gender stereotypes, transgender people encounter different degrees of negative experiences. These negative experiences exacerbate transgender women's survival difficulties, unemployment rate, and economic problems and further lead to the occurrence of commercial sex behavior, substance abuse, and other high-risk behavior [17]. In Wang et al.'s research, involvement in sex work among highly lonely transgender women who take drugs is three times that of others [18]. Compared with cisgender individuals, transgender individuals need more help to reduce drug use [19]. A combination of factors contributes to mental health problems among transgender women.

In Shandong province, China, there have been no studies on the psychological factors affecting transgender women. In this study, we investigated the psychological health status of transgender women in Shandong province, China, and explored the factors affecting their psychological health status.

## Methods

This study was a cross-sectional survey of the psychological distress of transgender women in Shandong, China. There is a population of more than 100 million in Shandong province, China, accounting for 7.19% of the national population, and its economy is developed. Its gross domestic product (GDP) was the third largest in China. However, the years of education for people over 15 years old is lower than the national average.

### Participants

There were 500 transgender women from Qingdao, Yantai, Linyi and Weifang in Shandong province who participated in this study from March 2021 to August 2021. The population of transgender women belongs to the hidden population, and it is difficult to obtain samples. Therefore, we used a combination of personal recommendation and convenience sampling to recruit the sample of transgender women [20]. First, the fixed bars, baths, hotels and massage parlors, which are the most prone to agglomeration, were selected as survey sites by transgender women within a certain range in each city. A survey was conducted by personal recommendation for transgender women who did not frequently enter these places. Transgender women who were unwilling to disclose personal information or to leave their familiar places of residence were met by investigators in the places where the subjects were located. Based on the eigenvalues obtained from the preliminary investigation, we estimated the initial sample size to be 329. To reduce the error caused by convenience sampling, we increased the sample size by more than 50%.

The exclusion criteria were as follows: individuals (1) who were not or who were unsure whether they were transgender women; (2) whose age was less than18 years old even though living or working in the local area; (3) whose answers were untrue or vague; (4) who had expressive disorder; or (5) who refused to sign an informed consent form.

In this study, 498 transgender women (*N* = 498) participated in the analysis, and participants ranged in age from 19 to 57 years, and all of them were Han people.

### Data collection

A preliminary structured questionnaire was formed by consulting numerous relevant studies and experts in related fields. We conducted a presurvey before the formal investigation. Through the feedback of volunteers and transgender women among the presurvey, the questionnaire was repeatedly modified to ensure that it was easy for respondents to understand and effective in the survey process. The questionnaire was composed of sociodemographic characteristics, AIDS-related knowledge, related sexual behaviors, substance abuse behaviors, psychological health and social support. Data were collected by the researchers themselves and volunteers from LGBT (lesbian, gay, bisexual, and transgender) members of social organizations. Prior to data collection, volunteers were trained on how to properly conduct one-on-one interviews to ensure that standardized, consistent data were available. Before the survey, all participants completed informed consent forms. (Additional file 1).

In the research of the LGBT population, many researchers have adopted the methods of online recruitment and self-report [11, 13, 21], while in this study, we used face-to-face interviews and filled out paper questionnaires to ensure quality. After the questionnaire was completed, participants received a free HIV/AIDS syphilis test provided by us. Among the 500 questionnaires collected, 498 were completed effectively after checking the completeness and consistency of the questionnaires and the qualified questionnaires were coded, and the questionnaire efficiency rate was 99.6%. To ensure the accuracy of the data, double entry was performed.

### Measures

#### 12-item General Health Questionnaire (GHQ-12)

For psychological distress, the 12-item General Health Questionnaire (GHQ-12) was used. The GHQ is one of the most widely used questionnaires for screening mental disorders and has been verified by many studies and has good reliability and validity. The English version has been translated into many languages and used in countries with different cultural backgrounds [22, 23]. There are 60-, 30-, 28-, 20- and 12-items of versions of the general health questionnaire [24]. The 12-item General Health Questionnaire (12-item General Health Questionnaire, GHQ-12) was used in this study. It has been verified many times and is one of the most commonly used measurement tools for mental health problems. It has the advantages of simplicity, time savings and fast response.

According to the research of Yang [24], it has been verified that the Chinese version of Taiwan province and the GHQ-12 have good applicability in mainland people, and the screening efficiency of the GHQ is similar to that of the Symptom Check List-90 (SCL-90). Cronbach's α was 0.92 in this study. The scoring method used in this study was the GHQ standard scoring method [25, 26], that is, the 0–0-1–1 scoring method. There are 12 topics in the questionnaire. Each topic has four options. The first two items were chosen to score 0 points, and the remaining two were chosen to score 1 point. The maximum possible score is 12, with 3 points as the cutoff value, and a score higher than 3 is considered positive for psychological disorders. The higher the score, the more serious the psychological problem [27].

### Sociodemographic characteristics

Sociodemographic characteristics are used to describe the personal and socioeconomic situation of transgender women in the Chinese cultural background. In the study, we asked about age, marital status, place of birth, education, occupation, income and population mobility of transgender women. In terms of employment, transgender women who chose service industries were asked about their specific jobs, including cashiers, stage performers, liquor salespeople, bartenders, etc.

### AIDS related knowledge

AIDS-related knowledge was used to describe their understanding of AIDS [28]. The topic was "Is HIV infection visible from the outside?", "Do mosquito bites spread AIDS? ", "Will eating with HIV-infected people or patients spread AIDS? ", "Will transfusing blood with HIV infect or spread AIDS? ", "Can sharing syringes with people with HIV infect or spread AIDS? ", "Can children born to HIV-infected women be infected with AIDS? ", "Can the correct use of condoms reduce the spread of AIDS? ", "Do you have to bear legal responsibility for intentionally spreading AIDS?".Participants who answered all the questions correctly were considered qualified; that is, those who were qualified were considered to have a good knowledge of AIDS. Otherwise, they were unqualified. Each question received 1 point, with a total score of 8 points. The above topics of AIDS-related knowledge are numbered from 1 to 8 in turn.

### Social Support Rate Scale (SSRS)

For social support, the social support rate scale (SSRS) developed by Xiao Shuiyuan in 1986 was selected. The SSRS has been proven to be very suitable for the Chinese population many times. In addition, the SSRS has the advantages of convenience and time savings, and its validity is relatively good [14]. The scale consisted of 10 items, with total score consistency *r* = 0.92 and item consistency between 0.89–0.94 [14]. A higher total SSRS score reflects a higher degree of social support. Generally, a total score below 20 indicates less social support, 20–30 points indicates general social support, and 30–40 points indicates that the degree of social support is relatively satisfactory.

### Data processing and analysis

EPIDATA 3.0 (Epidata association, Odense, Denmark) software was used to input data and establish a database, and STATA 14.0 (StataCorp LLC, College Station, Texas, USA) was used to organize and analyze the data. Logistic regression analysis was used to analyze the influence of different factors on psychological distress, and the variables with statistical significance in the single factor analysis were included in the multivariate logistic regression model for further analysis. The reason why we chose the univariate logistic rather than chi-square test was because univariate logistic regression offered more content than the chi-square test, and the results were consistent, such as crude OR [29]. Additionally, the Pearson chi-square value in the chi-square test was consistent with the chi-square score in binary logistic regression analysis.

Before multivariate analysis, we tested the collinearity among variables and found that the variance inflation factor (VIF) of each variable was less than 10. In univariate logistic regression results, variables with *p* < 0.20 were incorporated into the multivariate analysis model. All comparisons were bilateral, and *p* < 0.05 was considered to be statistically significant in multivariate logistic regression.

## Results

### GHQ—12 positive rate

The critical value of GHQ-12 positive rate is 3. If the score is greater than or equal to 3, the psychological state is negative. A negative psychological state is a positive result (psychological distress), that is, there may be a psychological problem. In 498 participants, 1 in 5 transgender women suffered from psychological distress (20.08%), and the average GHQ-12 score was 1.33 (SD = 1.47).

### Factors influencing psychology by social demographic characteristics

Among 498 participants, the mean age was 29.58 years (SD=5.73). Table 1 shows the number and proportion of transgender women who had psychological distress in terms of sociodemographic characteristics. Almost one quarter (22.7%) of unmarried transgender women had psychological distress compared with only 14.3% among those who were married, and only 6.7% among those who were divorced or widowed. Regarding the province of origin of the transgender women, 18.7% of those from Shandong had psychological distress compared with 22.2% among transgender women from other provinces. Two fifths (39.8%) of transgender women were from other provinces which indicated that they were highly mobile. During the investigation, according to the interviewees' self-report, in addition to the relatively strong mobility of transgender women engaged in relevant stage performances, some participants engaged in sexual services across provinces or cities according to the needs of customers. In terms of transgender women’s residence and occupation, there was a higher prevalence of distress among those living in towns and cities (around 20%) compared with those living in rural areas (15.3%) and among those working in the service industry (23.5%) compared with those in other occupations (10.9%).

The service industry included waiters, liquor salesmen, performers, cashiers, and sex workers, and other occupations included administrators, teachers, soldiers and students. Considerably lower prevalence among those with tertiary education. The univariate logistic regression results showed that education was a factor related to psychological distress. There was an inverse relationship between monthly income and prevalence of distress.

The result of univariate logistic regression analysis showed that individuals aged between 30 and 39 years (*OR*:0.37, 95% CI:0.0.22-0.60) and individuals over 39 years of age (*OR*:0.17, 95% CI:0.05-0.57) were less likely to have psychological distress than transgender women 18-29 years of age. In addition, transgender women who were divorced or widowed (*OR*: 0.24, 95% CI: 0.09-0.69) were less likely to have psychological distress than those who were unmarried. Transgender women who were engaged in occupations outside the service industry (*OR*:0.40, 95% CI:0.22-0.72) and those who earned more than 15,000 yuan (*OR*:0.54, 95% CI:0.04-0.87) were less likely to have psychological problems (Table 1).

**Table 1:** Univariate logistic regression analysis of sociodemographic characteristics related to transgender women's psychological distress

Variables	Total *N* (%)	Psychological distress *N* (%)	OR	*p*-value	95%CI	
Age						
18–29	248 (49.8)	71 (28.6)	1			
30–39	203 (40.8)	26 (12.8)	0.37	** < 0.001**	0.22	0.60
> 39	47 (9.4)	3 (6.4)	0.17	**0.004**	0.05	0.57
Marriage						
Unmarried	396 (79.5)	90 (22.7)	1			
In marriage	42 (8.4)	6 (14.3)	0.57	0.214	0.23	1.39
Divorced or widowed	60 (12.0)	4 (6.7)	0.24	**0.008**	0.09	0.69
Population mobility						
This province	300 (60.2)	56 (18.7)	1			
Other provinces	198 (39.8)	44 (22.2)	1.25	0.333	0.80	1.94
Residence						
Rural	85 (17.1)	13 (15.3)	1			
Township	150 (30.1)	30 (20.0)	1.39	0.371	0.68	2.83
City proper	263 (52.8)	57 (21.7)	1.53	0.205	0.79	2.96
Occupation						
Service industry	361 (72.5)	85 (23.5)	1			
Other	137 (27.5)	15 (10.9)	0.40	**0.002**	0.22	0.72
Education						
Primary or junior	115 (23.1)	26 (22.6)	1			
High school or technical secondary school and professional college	346 (69.5)	72 (20.8)	0.90	0.683	0.54	1.50
Undergraduate and postgraduate	37 (7.4)	2 (5.41)	0.20	**0.032**	0.04	0.87
Monthly Income						
< 5000	71 (14.3)	18 (25.4)	1			
5000–10,000	271 (54.4)	61 (22.5)	0.86	0.613	0.47	1.57
10,000–15,000	123 (24.7)	19 (15.4)	0.54	**0.094**	0.26	1.11
> 15,000	33 (6.6)	2 (6.1)	0.20	**0.033**	0.04	0.87

*OR*  O*dds ratio, CI*  C*onfidence interval*

### AIDS-related knowledge

The AIDS-related knowledge level was used to represent cognition on AIDS. The correct rate of “HIV-infected women will give birth to children infected with AIDS?” was the lowest (90.16%), and the correct rate of “Is there any legal responsibility for deliberately spreading AIDS?” and “Will transfusing blood with HIV infect or spread AIDS?” was highest (99%). The correct rate of each topic is shown in Supplementary Fig. 1.

Univariate logistic regression analysis indicated that the AIDS-related cognitive status was not significant (*p* > 0.2) and AIDS-related cognitive status was not a related factor of psychological stress. (Table 2).

**Table 2:** Univariate logistic regression of AIDS-related cognition, sexual behavior in six months, substance abuse, social support and STIs

Variables	Total N (%)	Psychological distress N (%)	OR	*p*-value	95%CI	
AIDS related cognition						
Qualified	130 (26.1)	70 (53.8)	1			
Unqualified	368 (73.9)	30 (7.9)	1.28	0.322	0.48	1.27
Times of anal sex in the last week						
< 5	333 (66.9)	69 (20.7)	1			
5–10	132 (26.5)	27 (20.4)	0.98	0.949	0.60	1.62
> 10	33 (6.6)	4 (12.1)	0.53	0.245	0.18	1.55
Frequency of anal condom wearing						
Every time	281 (56.4)	68 (24.2)	1.85	**0 .010**	1.16	2.94
Other	217 (43.6)	32 (14.7)	1			
Oral sex condom frequency						
Every time	51 (10.2)	19 (37.2)	2.68	**0 .002**	1.45	4.97
Other	447 (89.8)	81 (18.1)	1			
Number of sexual partners						
< 3	16 (3.2)	0	1			
3–5	41 (8.2)	7(17.1)	0.77	0.545	0.33	1.79
> 5	441 (88.5)	93 (21.1)	1			
Drinking alcohol						
Yes	271 (54.4)	54 (19.9)	0.98	0.925	0.63	1.52
No	227 (45.6)	46 (20.3)	1			
Drug using						
Yes	187 (37.5)	48 (25.7)	1.72	**0.016**	1.10	2.68
No	311 (62.4)	52 (16.7)	1			
Sexually transmitted infections (STIs)						
Yes	182 (36.5)	22 (12.1)	0.42	**0.001**	0.25	0.70
No	316 (63.4)	78 (24.7)	1			
Total Score for Social Support						
< 20	40 (8.0)	9 (22.5)	1.56	0.280	0.70	3.49
20–30	165 (33.1)	45 (27.3)	2.01	**0.003**	1.27	3.21
> 30	293 (58.8)	46 (15.7)	1			

*OR*  O*dds ratio, CI*  C*onfidence interval*

### Related sexual behavior, substance abuse and social support

From the number of anal intercourse in the previous week, only 12.1% of transgender women who had anal sex more than ten times last week had psychological distress compared with around 20% among those having anal sex less frequently. Among the transgender women who wore condoms during each anal sex encounter, 24.2% had psychological distress, compared with only 14.7% among those who did not consistently wear condoms during anal intercourse. In terms of frequency of oral sex condom use, 37.2% of transgender women who wore condoms every time reported psychological distress, while only 18.1% of the others did so. Transgender women who had fewer than three sexual partners within six months had no psychological distress, while 17.1% of transgender women with three to five sexual partners had psychological distress, and 21.1% of transgender women with more than five sexual partners within six months had psychological distress.The prevalence of psychological stress was similar among transgender women who drank and did not drink alcohol but was higher among those who used drugs (25.7%) than among those who did not (16.7%). In addition, we also included a history of sexually transmitted infections (STIs) other than AIDS in the univariate logistic regression analysis. Only 12.1% of transgender women with STIs had psychological distress, while 24.7% of transgender women without STIs had psychological distress. The average score for social support was 33 (SD = 9.67). Among transgender women with insufficient and general social support, 22.5% and 27.3% had psychological distress, while among transgender women reporting high social support, only 15.7% had psychological distress. We found that using condoms every time during anal sex (*OR*:1.85, 95% CI:1.16–2.94), using condoms every time during oral sex (*OR*:2.68, 95% CI:1.45–4.97), drug use (*OR*:1.72, 95% CI:1.10–2.68), STIs (*OR*:0.42, 95% CI:0.25–0.70), and scores between 20 and 30 on social support (*OR*:2.01, 95% CI:1.27–3.21) were all factors related to the psychological distress of transgender women. (Table 2).

### The relation of gender identity and adolescent experience with psychological distress

We also performed some analysis on gender identity and the psychological impact of adolescent experiences on transgender women. These aspects were reflected in some problems, and were also included in the univariate logistic regression analysis, with the specific topic as follows: “Did you ever think that being a girl was better than being a boy when you were a teenager?”, “Did you particularly like to play with boys when you were child?”, “Do you agree with your own sexual orientation?”, “Have you made your sexual orientation and identity public?”, “Do you want to be recognized for your sexual orientation and identity?”, “Do you think the people around you think differently about you?” and “Has the homosexual culture on the internet helped you determine your sexual orientation?”.

The univariate logistic regression analysis showed that the factors that related to psychological distress. Compared to those who thought that being a girl was better than being a boy at an early age, those who did not have such thoughts were less likely to have psychological distress (*OR*: 0.33, 95% CI: 0.18–0.62). Other characteristics of transgender women with lower prevalence of psychological distress were: never disclosing their sexual orientation and identity, or only disclosing to friends and family (*OR*: 0.22, 95% CI: 0.14–0.40, and *OR*: 0.22, 95% CI: 0.10–0.48, respectively); and not feeling having strange eyes around (OR: 0.28, 95% CI: 0.15–0.51). On the contrary, there were other factors that might increase the risk of psychological distress of transgender women, which included not being raised as a girl at an early age (*OR*:1.41, 95%CI:0.90–2.23), not desiring to be identified with one's sexual orientation and identity (*OR*:9.61, 95% CI:5.03–18.35), and the Internet not playing an essential role in helping determine sexual orientation (*OR*:12.17, 95% CI:7.09–20.93). (Table 3).

**Table 3:** Results of univariate logistic regression of gender identity and adolescent experience on psychological distress

Variables	Total *N* (%)	Psychological distress *N* (%)	OR	*p*-value	95%CI	
Raised as a girl at an early age						
Yes	207 (41.6)	35 (16.9)	1			
No	291 (58.4)	65 (22.3)	1.41	**0.140**	0.90	2.23
Been a girl is better than being a boy at an early age						
Yes	362 (72.7)	87(24.0)	1			
No	136 (27.3)	13 (9.6)	0.33	**0.001**	0.18	0.62
Encountered violence in school as a teenager						
Yes	167 (33.5)	33 (19.8)	1			
No	331 (66.5)	67 (20.2)	1.03	0.899	0.65	1.64
Liked to play with boys at an early age						
Yes	437 (87.7)	89 (20.4)	1			
General	41 (8.2)	10 (24.4)	1.26	0.544	0.60	2.67
No	20 (4.0)	1 (5.0)	0.21	**0.126**	0.03	1.56
Sexual orientation identity						
Yes	387 (77.7)	75 (19.4)	1			
General	104 (20.9)	23 (22.1)	1.18	0.536	0.70	2.00
No	7 (1.4)	2 (28.6)	1.66	0.547	0.32	8.74
Keep one's romance a secret						
Yes	165 (33.2)	37 22.4)	1			
No	332 (66.8)	63 (19.0)	0.79	0.325	0.50	1.26
Disclosure of sexual orientation and identity						
Yes	72 (14.5)	10 (13.9)	1			
Only friends and families knew	332 (66.7)	50 (15.1)	0.24	** < 0.001**	0.14	0.40
No	94 (18.9)	40 (42.6)	0.22	** < 0.001**	0.10	0.48
Desiring to be identified with one's sexual orientation and identity						
Yes	451 (90.6)	70 (15.5)	1			
No	47 (9.4)	30 (63.8)	9.61	** < 0.001**	5.03	18.35
Feel around have strange eyes						
Yes	337 (67.7)	86 (25.5)	1			
No	161 (32.3)	14 (8.7)	0.28	** < 0.001**	0.15	0.51
The internet helped determine sexual orientation						
Yes	315 (63.2)	29 (9.2)	1			
Not essential	105 (21.1)	58 (55.2)	12.17	** < 0.001**	7.09	20.93
No	78 (15.7)	13 (16.7)	1.97	0.060	0.97	4.00

*OR*  O*dds ratio, CI*  C*onfidence interval*

### Predictors of transgender women's psychology

Significant variables in univariate logistic regression were incorporated into the multivariate logistic regression model (*p* < 0.20). Multivariate logistic regression analysis showed participants who earned a monthly income between 10,000–15,000 yuan (*OR*: 0.16, 95% CI:0.06–0.45) or greater than 15,000 yuan (*OR*: 0.07, 95% CI:0.01–0.43) were less likely to have psychological distress. Additionally, transgender women who never disclosed their sexual orientation and identity (*OR*: 0.19, 95% CI: 0.06–0.58), who only disclosed their sexual orientation and identity to families and friends (*OR*: 0.41, 95% CI: 0.18–0.93) were also less likely to have psychological distress. By contrast, transgender women who did not desire to be identified with their sexual orientation and identity (*OR*: 3.31, 95% CI: 1.08–10.16) and who reported that the Internet did not play an essential role in helping determine sexual orientation (*OR*: 5.96, 95% CI: 2.91–12.20) were more likely to have psychological distress. (Table 4).

**Table 4:** Predictors of transgender women's psychological distress in multivariate logistic regression

Variables	OR	*p*-value	95%CI	
Age				
18–29	1			
30–39	0.72	0.311	0.38	1.37
> 39	0.62	0.507	0.15	2.55
Marriage				
Unmarried	1			
In marriage	0.64	0.414	0.21	1.88
Divorced or widowed	0.75	0.649	0.22	2.54
Occupation				
Service industry	1			
Other	0.47	0.058	0.22	1.02
Education				
Primary or junior	1			
High school or technical secondary school and Professional college	0.92	0.798	0.47	1.80
Undergraduate and postgraduate	0.31	0.179	0.05	1.72
Monthly Income				
< 5000	1			
5000–10,000	0.47	0.076	0.20	1.08
10,000–15,000	0.16	** < 0.001**	0.06	0.45
> 15,000	0.07	**0.004**	0.01	0.43
Frequency of anal condom wearing				
Every time	1.07	0.850	0.52	2.23
Other	1			
Oral sex condom frequency				
Every time	1.88	0.179	0.75	4.71
Other	1			
Drug using				
Yes	0.77	0.426	0.41	1.46
No	1			
Sexually transmitted infections (STIs)				
Yes	0.62	0.156	0.32	1.20
No	1			
Total score for social support				
< 20	1.52	0.475	0.48	4.78
20–30	1.70	0.176	0.79	3.67
> 30	1			
Raised as a girl at an early age				
Yes	1			
No	0.85	0.618	0.45	1.61
Been a girl is better than being a boy at an early age				
Yes	1			
No	0.51	0.085	0.23	1.10
Liked to play with boys at an early age				
Yes	1			
General	0.78	0.638	0.28	2.18
No	0.27	0.227	0.03	2.27
Disclosure of sexual orientation and identity				
Yes	1			
Only friends and families knew	0.41	**0.033**	0.18	0.93
No	0.19	**0.004**	0.06	0.58
Desiring to be identified with one's sexual orientation and identity				
Yes	1			
No	3.31	**0.037**	1.08	10.16
Feel around have strange eyes				
Yes	1			
No	0.55	0.159	0.24	1.26
The internet helped determine sexual orientation				
Yes	1			
Not essential	5.96	** < 0.001**	2.91	12.20
No	1.40	0.451	0.58	3.40

*OR*  O*dds ratio, CI*  C*onfidence interval*

## Discussion

To the best of our knowledge, this study is the first to use the GHQ-12 in Shandong province, China, to investigate the psychological factors affecting transgender women. In this study, we added a new variable of high-risk sexual behavior, that is, the frequency of oral condom use. Among other psychologically related factors, we also included the influence of the internet on transgender women. The majority of participants were young people under 39 years old (90.6%). A previous study showed that the psychology of transgender women was related to their age, and participants of different ages showed obvious mental health differences [30]. In our univariate logistic regression study, younger age was one of the factors related to the psychological distress of transgender women. Among the transgender women, a majority were working in the service industry (72.5%). Some of the transgender women who were unemployed would selectively serve or be kept by a client. In addition, the mobility of transgender women in this study was relatively strong, with 39.8% of nonlocal transgender women [31]. Although this study did not find a relationship between the mobility of transgender women and their psychological distress, factors such as the high mobility of transgender women, the burden of the HIV epidemic and access to resources deserve further study [32].

Related studies have shown that the differences between urban and rural areas are related to the psychological state of LGBT individuals [33, 34]. There are many reasons for the association, which may be because transgender women living in cities and towns can access public services more easily and are more vulnerable to discrimination and pressure from the outside [35, 36]. However, we did not find an association between psychological distress and residence in our analysis (*p* > 0.2).

Sex services and high-risk sex behaviors were common among transgender women, and sex work has been reported to be associated with more stigma and discrimination [37]. On the basis of the description of the transgender women surveyed, they were engaged in general service industries at the same time, such as performance and beverage sales, and also performed sex services part-time. Some held part-time jobs in the general service industry, while sex work was their main occupation. Although occupation of transgender women was a factor associated with psychological distress in univariate logistic regression, occupation was not significant in multivariate logistic regression. For transgender women who had psychological distress, their perception of unprotected sexual behavior may be affected [38]. At present, research on psychological distress and high-risk sexual behavior generally includes the influence of psychological status on high-risk sexual behavior, or whether sexual abstinence and risky sex are associated with psychological symptoms [39, 40]. In our analysis, we regarded high-risk sexual behavior as a factor related to the psychology of transgender women. When asked whether they wear condoms during anal sex, only 56.4% of transgender women reported wearing condoms every time they had sex. We found that the frequency of wearing condoms during anal sex was an important factor related to the psychological distress of transgender women in the univariate logistic regression analysis. In the survey, we learned that some transgender women engaged in sexual services, and those transgender women gave up wearing condoms because customers provided more money or other benefits. However, in the multivariate model, the frequency of anal condom use was not significant. Similarly, the frequency of oral condom use was significant in univariate logistic regression but when included in multivariate logistic regression it was no longer significant. Even so, since there are few studies on oral sex, especially among transgender women, the oral sex behavior of trans women deserves further research. In our survey, according to the statements of most transgender women, who believed that it was almost impossible to transmit AIDS or other sexually transmitted diseases through oral sex, all generally chose not to wear condoms. They thought that unless their oral cavity was damaged, they could not contract AIDS. On the other hand, they believed that this would affect the comfort of oral sex. A study on oral sex among transgender youth pointed out that although the surveyed youth had some knowledge about oral sex, they chose not to wear a condom because they did not want to wear them, they thought condoms felt uncomfortable or they did not think they needed to wear them [41]. The frequency of wearing condoms during oral sex was very low, and only 10.2% of transgender women chose to wear condoms every time they engaged in oral sex. Of the transgender women who wore condoms every time they had oral sex, 37.2% reported psychological distress. Therefore, it is still an important aspect to formulate individualized intervention measures, especially to improve the communication between transgender women and partners who choose to wear condoms during sexual intercourse.

The monthly income of transgender women was a factor that affected the psychological distress of transgender women. Compared to transgender women with a monthly income of less than 5000 yuan, transgender women with an income of 10,000–15,000 (OR: 0.16, 95% CI: 0.06–0.45) and with an income of more than 15,000 (OR: 0.07, 95% CI: 0.01–0.43) were less likely to have psychological distress. A study from the US found that transgender people with low incomes report more psychological distress [42]. A study in Taiwan province showed that LGBT groups with incomes above NT$ 5,000 (equivalent to 4,500 yuan) had better physical health, psychological health, social relationships and environmental health [43]. This portion of high-income transgender women might be due to the improvement in the quality of life brought about by the improvement in economic conditions while increasing the sense of security in personal life.

Transgender women in China are a vulnerable group. Due to their traditional cultural background, they receive less social support. Many transgender women found that it was difficult to choose to disclose their identity and obtain social support. Legitimate gender identity may improve the psychological status of transgender women [44, 45]. Inconsistency in identity and behavior can lead to adverse health consequences, including psychological problems such as anxiety, depression, suicide, drug abuse, and high-risk sexual behaviors [42, 46]. Sexual orientation is also risk factor for the spread of AIDS [47]. Compared with disclosing their sexual orientation and identity, transgender women who did not disclose their sexual orientation (*OR*: 0.19, 95% CI: 0.06–0.58) and identity or only disclosed their sexual orientation and identity to families and friends (*OR*: 0.41, 95% CI: 0.18–0.93) were less likely to have psychological distress. Due to the existence of the traditional concept of dual gender, they were afraid of discrimination brought by different views, causing their anxiety symptoms [48]. The research on the gender identity of transgender women has always been a problem worth paying attention to. In a qualitative study of transgender women in Jiangsu province, China, most transgender women only tended to reveal their gender identity to family members or close friends, but generally, in the family, they will be rejected by their family and may be subjected to violence or abuse [49]. The perception of strange looks around represented the discrimination transgender women felt themselves. However, this variable was not significant in multivariate logistic regression. In determining gender and sexual orientation among transgender women, the Internet was playing an increasingly important role, which also had a certain impact on the psychology of transgender women. The rapid development of the internet has provided transgender women with information they need. A study was conducted to verify the effectiveness of internet intervention in young people's healthy behaviors [50]. Sexual minorities were more likely to access health information on the internet [51],and they interacted with the internet to confirm their sexual identity [52]. Compared with the idea that the internet was helpful for transgender women in confirming their sexual orientation and gender, transgender women who believed that the Internet played a non-essential role in helping determine orientation (*OR*:5.96, 95%CI:2.91–12.20) were more likely to experience psychological distress.

This article is not without limitations. In fact, we stipulate only the identity of transgender women without stipulating the sexual orientation of transgender women. They may be heterosexual, gay or bisexual, which may affect the reaction of transgender women to a certain extent. Our research is a cross-sectional study, which cannot reflect a causal relationship between predictors and variables. Because the sampling method was nonrandom, there may be selection bias. In addition, this study was only conducted among transgender women in Shandong province, China, which may be different from other regions due to the influence of cultural background. Finally, due to the particularity of the GHQ-12 scale, we did not use its dimension, but in the future, we can further verify the dimension problem of the GHQ-12 scale in the population of transgender women and expand the application of the scale.

## Conclusion

1 in 5 transgender women suffered from psychological distress (20.08%). As a vulnerable group, transgender women are prone to psychological distress. In summary, the GHQ-12 was used to measure the psychological factors affecting transgender women in Shandong, China. Having a higher income were better for transgender women’s psychological health.

Although the included variables of high-risk sexual behavior were not predictors of the psychological distress of transgender women, an association was found in univariate logistic regression analysis. Transgender women who chose to come out were more likely to report psychological distress than those who refused to disclose their sexual orientation and gender. Improving the internet's understanding of transgender women's gender, sexual orientation and role will help to spread common knowledge about transgender women and further improve the social acceptance of transgender women. Therefore, we suggest formulating appropriate strategies combined with the internet to influence the sexual behavior of transgender women, improve their social understanding and pay attention to their psychological health.

## Supplementary Information

**Additional file 1.** 

## Data Availability

The datasets used and/or analyzed during the current study are available from the corresponding author on reasonable request.

## Abbreviations

GHQ-12: 12-Item General Health Questionnaire
AIDS: Acquired Immune Deficiency Syndrome
MSM: Men who have sex with men
HIV: Human Immunodeficiency Virus
SSRS: Social support rate scale
LGBT: Lesbian, gay, bisexual and transgender
LR: Likelihood ratio
STIs: Sexually transmitted infections
Std. Err: Standard error
OR: Odds ratio
CI: Confidence interval
SD: Standard deviation

## References

[1] Schulman JK, Erickson-Schroth L (2017). Mental Health in Sexual Minority and Transgender Women. Psychiatr Clin North Am.

[2] Poteat T, Wirtz AL, Radix A, Borquez A, Silva-Santisteban A, Deutsch MB, Khan SI, Winter S, Operario D (2015). HIV risk and preventive interventions in transgender women sex workers. Lancet (London, England).

[3] Cai Y, Wang Z, Lau JT, Li J, Ma T, Liu Y (2016). Prevalence and associated factors of condomless receptive anal intercourse with male clients among transgender women sex workers in Shenyang, China. J Int AIDS Soc.

[4] Baral SD, Poteat T, Strömdahl S, Wirtz AL, Guadamuz TE, Beyrer C (2013). Worldwide burden of HIV in transgender women: a systematic review and meta-analysis. Lancet Infect Dis.

[5] Wenjing X. Mental Health, Behavioral Characteristics and HIV Prevalence of Transgender Women in Jiangsu Province. *Mster.* Nanjing Medical University, China; 2020. 10.27249/d.cnki.gnjyu.2020.000242.

[6] Meyer IH (1995). Minority stress and mental health in gay men. J Health Soc Behav.

[7] Russell JS, Hickson DA, Timmins L, Duncan DT. Higher Rates of Low Socioeconomic Status, Marginalization, and Stress in Black Transgender Women Compared to Black Cisgender MSM in The MARI Study. Int J Environ Rese Public Health. 2021;18(4):2183. 10.3390/ijerph18042183 PMC7927022 33672272

[8] Fontanari AMV, Rovaris DL, Costa AB, Pasley A, Cupertino RB, Soll BMB, Schwarz K, da Silva DC, Borba AO, Mueller A (2018). Childhood Maltreatment Linked with a Deterioration of Psychosocial Outcomes in Adult Life for Southern Brazilian Transgender Women. J Immigr Minor Health.

[9] Streed CG, McCarthy EP, Haas JS (2018). Self-Reported Physical and Mental Health of Gender Nonconforming Transgender Adults in the United States. LGBT health.

[10] Carmel TC, Erickson-Schroth L (2016). Mental Health and the Transgender Population. J Psychosoc Nurs Ment Health Serv.

[11] Veale JF, Watson RJ, Peter T, Saewyc EM (2017). Mental Health Disparities Among Canadian Transgender Youth. The Journal of adolescent health : official publication of the Society for Adolescent Medicine.

[12] Zucchi EM, Barros C, Redoschi BRL, Deus LFA, Veras M (2019). Psychological well-being among transvestites and trans women in the state of São Paulo, Brazil. Cad Saude Publica.

[13] Budge SL, Adelson JL, Howard KA (2013). Anxiety and depression in transgender individuals: the roles of transition status, loss, social support, and coping. J Consult Clin Psychol.

[14] Xiao S (1994). Theoretical basis and research application of Social Support Rating Scale. J Clin Psychiatry.

[15] Nuttbrock LA, Hwahng SJ (2017). Ethnicity, Sex Work, and Incident HIV/STI Among Transgender Women in New York City: A Three Year Prospective Study. AIDS Behav.

[16] Howell J, Maguire R (2019). Seeking help when transgender: Exploring the difference in mental and physical health seeking behaviors between transgender and cisgender individuals in Ireland. The international journal of transgenderism.

[17] Xia N, Liu A (2021). A review of mental health in transgender people. Chin Ment Health J.

[18] Wang Q, Chang R, Wang Y, Jiang X, Zhang S, Shen Q, Wang Z, Ma T, Lau JTF, Cai Y (2020). Correlates of alcohol and illicit drug use before commercial sex among transgender women with a history of sex work in China. Sexual health.

[19] Connolly D, Davies E, Lynskey M, Barratt MJ, Maier L, Ferris J, Winstock A, Gilchrist G (2020). Comparing intentions to reduce substance use and willingness to seek help among transgender and cisgender participants from the Global Drug Survey. J Subst Abuse Treat.

[20] Setia MS (2016). Methodology Series Module 5: Sampling Strategies. Indian J Dermatol.

[21] Garthe RC, Hidalgo MA, Hereth J, Garofalo R, Reisner SL, Mimiaga MJ, Kuhns L (2018). Prevalence and Risk Correlates of Intimate Partner Violence Among a Multisite Cohort of Young Transgender Women. LGBT health.

[22] Ip WY, Martin CR (2006). Psychometric properties of the 12-item General Health Questionnaire (GHQ-12) in Chinese women during pregnancy and in the postnatal period. Psychol Health Med.

[23] Romppel M, Braehler E, Roth M, Glaesmer H (2013). What is the General Health Questionnaire-12 assessing? Dimensionality and psychometric properties of the General Health Questionnaire-12 in a large scale German population sample. Compr Psychiatry.

[24] Yang Tingzhong H (2003). Wu Zhenyi: The application of Chinese health questionnaire for mental disorder screening in community settings in mainland China. Chinese Journal of Epidemiology.

[25] Wang Wen-qiang DL-j LZ-h (2012). Wen Cheng, Hong Xu, Chen Ying, Wang Yu-zhen, Li Xiu-zhuo, Zhou Jian-qing: The best thresholds the screening features among the three scoring methods of the 12-items General Health Questionnaire. Chinese Journal of Psychiatry.

[26] Graetz B (1991). Multidimensional properties of the General Health Questionnaire. Soc Psychiatry Psychiatr Epidemiol.

[27] Wang JLYW, Zhang HL, Wang XC (2019). Study on the general mental health status and influencing factors of Chinese Medical Team members. Chinese Journal of Epidemiology.

[28] Cai Y, Shi R, Li S, Xu G, Huang H (2012). Study of HIV/AIDS-related knowledge among junior high-school students in Shanghai, China. Int J STD AIDS.

[29] Deping MGL (2012). Logistic regression analysis and SAS implementation in medical research.

[30] Turner CM, Arayasirikul S, Wilson EC (1982). Disparities in HIV-related risk and socio-economic outcomes among trans women in the sex trade and effects of a targeted, anti-sex-trafficking policy. Soc Sci Med.

[31] Glick JL, Lopez A, Pollock M, Theall KP (2019). "Housing Insecurity Seems to Almost Go Hand in Hand with Being Trans": Housing Stress among Transgender and Gender Non-conforming Individuals in New Orleans. Journal of urban health : bulletin of the New York Academy of Medicine.

[32] Goedel WC, Regan SD, Chaix B, Radix A, Reisner SL, Janssen AC, Duncan DT. Using global positioning system methods to explore mobility patterns and exposure to high HIV prevalence neighbourhoods among transgender women in New York. Geospatial Health. 2019;14(2):351–56. 10.4081/gh.2019.752 PMC9897014 31724385

[33] Kauth MR, Barrera TL, Denton FN, Latini DM (2017). Health Differences Among Lesbian, Gay, and Transgender Veterans by Rural/Small Town and Suburban/Urban Setting. LGBT health.

[34] Austin EL (2013). Sexual orientation disclosure to health care providers among urban and non-urban southern lesbians. Women Health.

[35] Smith AJ, Hallum-Montes R, Nevin K, Zenker R, Sutherland B, Reagor S, Ortiz ME, Woods C, Frost M, Cochran B (2018). Determinants of Transgender Individuals' Well-Being, Mental Health, and Suicidality in a Rural State. Rural mental health.

[36] Ruben MA, Livingston NA, Berke DS, Matza AR, Shipherd JC (2019). Lesbian, Gay, Bisexual, and Transgender Veterans' Experiences of Discrimination in Health Care and Their Relation to Health Outcomes: A Pilot Study Examining the Moderating Role of Provider Communication. Health equity.

[37] Milner AN, Hearld KR, Abreau N, Budhwani H, Mayra Rodriguez-Lauzurique R, Paulino-Ramirez R (2019). Sex work, social support, and stigma: Experiences of transgender women in the Dominican Republic. The international journal of transgenderism.

[38] Dougan S, Elford J, Chadborn TR, Brown AE, Roy K, Murphy G, Gill ON (2007). Does the recent increase in HIV diagnoses among men who have sex with men in the UK reflect a rise in HIV incidence or increased uptake of HIV testing?. Sexually transmitted infections.

[39] Miller CT, Solomon SE, Bunn JY, Varni SE, Hodge JJ (2015). Psychological symptoms are associated with both abstinence and risky sex among men with HIV. Arch Sex Behav.

[40] Shacham E, Basta TB, Reece M (2009). Relationship of psychological distress and unprotected sex among individuals with HIV seeking mental health care.. Journal of the
International Association of Physicians in AIDS Care.

[41] Macdonald DW, Grossoehme DH, Mazzola A, Pestian T, Schwartz SB (2020). Oral Sex Knowledge and Experience of Transgender Youth: An Opportunity for Dental Education. J Dent Educ.

[42] Maksut JL, Sanchez TH, Wiginton JM, Scheim AI, Logie CH, Zlotorzynska M, Lyons CE, Baral SD (2020). Gender identity and sexual behavior stigmas, severe psychological distress, and suicidality in an online sample of transgender women in the United States. Ann Epidemiol.

[43] Wang YC, Chang SR, Miao NF (2021). Health Status and Quality of Life of Middle-Aged and Older Taiwanese Sexual and Gender Minorities. Journal of nursing scholarship : an official publication of Sigma Theta Tau International Honor Society of Nursing.

[44] Scheim AI, Perez-Brumer AG, Bauer GR (2020). Gender-concordant identity documents and mental health among transgender adults in the USA: a cross-sectional study. The Lancet Public health.

[45] Baldwin A, Dodge B, Schick V, Hubach RD, Bowling J, Malebranche D, Goncalves G, Schnarrs PW, Reece M, Fortenberry JD (2015). Sexual self-identification among behaviorally bisexual men in the midwestern United States. Arch Sex Behav.

[46] Olson-Kennedy J, Cohen-Kettenis PT, Kreukels BP, Meyer-Bahlburg HF, Garofalo R, Meyer W, Rosenthal SM (2016). Research priorities for gender nonconforming/transgender youth: gender identity development and biopsychosocial outcomes. Curr Opin Endocrinol Diabetes Obes.

[47] Nadal KL, Davidoff KC, Fujii-Doe W (2014). Transgender women and the sex work industry: roots in systemic, institutional, and interpersonal discrimination. Journal of trauma & dissociation : the official journal of the International Society for the Study of Dissociation (ISSD).

[48] Yang X, Wang L, Gu Y, Song W, Hao C, Zhou J, Zhang Q, Zhao Q (2016). A cross-sectional study of associations between casual partner, friend discrimination, social support and anxiety symptoms among Chinese transgender women. J Affect Disord.

[49] Yan ZH, Lin J, Xiao WJ, Lin KM, McFarland W, Yan HJ, Wilson E (2019). Identity, stigma, and HIV risk among transgender women: a qualitative study in Jiangsu Province, China. Infect Dis Poverty.

[50] Whiteley LB, Brown LK, Curtis V, Ryoo HJ, Beausoleil N (2018). Publicly Available Internet Content as a HIV/STI Prevention Intervention for Urban Youth. J Primary Prevent.

[51] Lee JH, Giovenco D, Operario D (2017). Patterns of Health Information Technology Use according to Sexual Orientation among US Adults Aged 50 and Older: Findings from a National Representative Sample-National Health Interview Survey 2013–2014. J Health Commun.

[52] Pingel ES, Bauermeister JA, Johns MM, Eisenberg A, Leslie-Santana M (2013). "A safe way to explore": Reframing risk on the Internet amidst young gay men's search for identity. J Adolesc Res.

