# Supplementary material for: The factors influencing the psychological distress of transgender women in Shandong, China: a cross-sectional study
Source: BMC Public Health. 2022 May 12;22:955. doi: 10.1186/s12889-022-13357-9 (PMC9101853; doi:10.1186/s12889-022-13357-9)
Supplement: Supplementary file 1 — Additional file 1. [file 12889_2022_13357_MOESM1_ESM.docx]

**Questionnaire on psychological health among transgender women in Shandong province**

Hello! We sincerely invite you to participate in this survey.We are the transgender women psychology research team of Dalian Medical University.The purpose of this study is to understand the psychological status of transgender women.You will fill out the questionnaire according to the content. This survey is anonymous, and we promise to keep your information and answers completely confidential. The data obtained will only be used for scientific research. The investigation is entirely voluntary.And,we hope your answer is true and that we can provide you with some assistance at the end of the investigation. We hope you will support our work.

Sincerely thank you for your support and cooperation, I wish you good health and happy life!

**Part I:Sociological demographic characteristics**

| A01 | Age: |
| --- | --- |
| A02 | Nationality: |
| A03 | Marital status:  ①Discoverture ②In marriage ③Divorced or widowed |
| A04 | Your domicile place:  ①This province ②Other provinces (Please specify province) |
| A05 | Your current residence ①Rural ②Township ③Urban |
| A06 | Your occupation:  ①Workers ②Farmers ③Administrative workers ④Service workers ⑤Students  ⑥Teachers ⑦[Individual business](http://www.baidu.com/link?url=7TJj54F43TFK34_6ZEXJd4dJajiueW0cXv7lfOhE9_RxPSQYPYLjoWK9c5ZXU15Ql6mCSjUtjRwHLCZyB-AuYP5smWaDSv3SYa5t3yb2f-E6RaJ03iwpqkLvUjeXnGy6) ⑧Military personnel ⑨Retired people  ⑩The unemployed |
| A07 | Your highest degree:  ①Primary school ②Junior middle school ③High school or secondary school  ④Junior college ⑤Bachelor degree ⑥Master degree or above |
| A08 | Your monthly income(yuan):  ①below 5000 ②5000-10000 ③10000-15000 ④15000 or more |

**Part II:AIDS related knowledge**

| B01 | If a person is infected with aids, can you tell from the appearance?  ①Yes ②No ③I don't know |
| --- | --- |
| B02 | Does mosquito bite spread AIDS?  ①Yes ②No ③I don't know |
| B03 | Will eating with HIV-infected people or patients spread or infect AIDS?  ①Yes ②No ③I don't know |
| B04 | Will inputting blood with HIV infect or spread AIDS?  ①Yes ②No ③I don't know |
| B05 | Can sharing injector with people living with HIV infect or spread AIDS?  ①Yes ②No ③I don't know |
| B06 | Can sharing injector with people living with HIV infect or spread AIDS?  ①Yes ②No ③I don't know |
| B07 | Correct use of condoms can reduce the spread of aids?  ①Yes ②No ③I don't know |
| B08 | Is there any legal responsibility for deliberately spreading AIDS?  ①Yes ②No ③I don't know |

**Part III:Sexual behavior**

**Anal sex**

| C01 | Have you had anal sex with someone of the same sex in the last six months?  ①Yes ②No ③Refuse to answer |
| --- | --- |
| C02 | How many times have you had anal sex with someone of the same sex in the last week?  ①times ②Refuse to answer |
| C03 | How often have you used condoms during anal sex with other men in the last six months?  ①Never used ②Sometimes used ③Every time ④Refuse to answer |
| C04 | How often have you used condoms during oral sex with other men in the last six months?  ①Never used ②Sometimes used ③Every time ④Refuse to answer |
| C06 | In the last six months, did you use a condom during your last sexual encounter with a man after drinking alcohol?  ①Yes ②No ③Don't drink ④Not having sex after drinking alcohol |

**Other behaviors**

| D01 | The number of sexual partners you've had in the last six months?  ①0 ②1-2 ③3-5 ④5 or more |
| --- | --- |

**Part IV:Survey of drug use behavior**

| E01 | Do you take drugs?  ①Yes ②No ③Refuse to answer |
| --- | --- |

**Part V:Investigation of diagnosis and prevention and control**

| F01 | Have you been diagnosed with a sexually transmitted disease in the past year?  ①Yes ②No ③Refuse to answer |
| --- | --- |

**Part VI:Social support**

| G01 | How many close friends do you have that you can count on for support and help?  ①0 ②1-2③3-5 ④6 or more | | | | |
| --- | --- | --- | --- | --- | --- |
| G02 | Over the past year,you:  ①live alone and far away from your family  ②often change places, and spend most of time with strangers  ③live with a classmate, colleague or friends  ④live with your family | | | | |
| G03 | You with your neighbors:  ①Never care about each other, just a nodding acquaintance  ②May be a little concerned about each other if you encounter the difficulties  ③Some of the neighbors are very concerned about you  ④Most of the neighbors care about you | | | | |
| G04 | You with your colleagues:①Never care about each other, just a nodding acquaintance  ②May be a little concerned about each other if you encounter the difficulties  ③Some of the colleagues are very concerned about you  ④Most of the colleagues care about you | | | | |
| G05 | Support and care received from family members (Tick the appropriate box "√") | | | | |
|  | options | None | Little | general | give one's full backing |
|  | a.Husband and wife (lover) |  |  |  |  |
|  | b.parents |  |  |  |  |
|  | c.children |  |  |  |  |
|  | d.brothers and sisters |  |  |  |  |
|  | e.Other members (such as sister-in-law) |  |  |  |  |
| G06 | In the past, when you were in a critical situation, you received financial support and assistance to solve practical problems from the following sources:  ①No source ②The following sources: (Optional)  a. Spouse b. Other family members c. Friends d. Relatives e. Colleagues  f. Work unit g. Official or semi-official organizations such as party, group and trade union  h. Non-official organizations such as religious and social organizations i. Other | | | | |
| G07 | In the past, you have had a source of comfort and concern in times of emergency:  ①No source ②The following sources: (Optional)  a. Spouse b. Other family members c. Friends d. Relatives e. Colleagues f. Work unit  g. Official or semi-official organizations such as party, group and trade union  h. Non-official organizations such as religious and social organizations i. Other | | | | |
| G08 | How do you talk about your troubles?  ①Never complain to anyone  ②Only complain to one or two very close people  ③If a friend asks you, you will tell them ④Take the initiative to talk about your troubles to get support and understanding | | | | |
| G09 | When you encounter trouble, how to ask for help:  ①Don't accept help from others ② Seldom ask others for help ③ Sometimes ask others for help ④Often ask for help from family, friends and organizations when you have difficulties | | | | |
| G10 | For groups (such as party organizations, religious organizations, trade unions, student unions and other organizational activities, you:  ①never participate ②sporadically participate ③frequently participate  ④actively participate and actively participate | | | | |

**Part VII:Psychological status**

**(please select the options that suit your situation in the past 3weeks)**

| H01 | Can you stay focused for the last three weeks?  ①Can concentrate ②As usual ③Can't concentrate ④Can't concentrate at all |
| --- | --- |
| H02 | Do you lose sleep worrying too much about something?  ①Never ②As usual ③Ever ④Always |
| H03 | Do you think you are a useful person in life?  ①Useful ②As usual ③Useless ④Completely useless |
| H04 | Do you feel able to make decisions about things that require decisions?  ①Can ②As usual ③Stressed ④Very stressed |
| H05 | Feeling stressed all the time?  ①No pressure ②As usual ③Under pressure ④Under great pressure |
| H06 | Do you think you can overcome difficulties in work/life?  ①Yes ②As usual ③No ④Not at all |
| H07 | Can you enjoy everyday life?  ①Yes ②As usual ③No ④Not at all |
| H08 | Do you feel able to face your problems?  ①Yes ②As usual ③No ④Not at all |
| H09 | Are you feeling down or depressed?  ①Don't feel ②As usual ③Feel ④Always feel |
| H10 | Are you lost faith in yourself?  ①Not ②As usual ③Lost ④Completely lost |
| H11 | Do you feel like a worthless person?  ①Don't feel ②As usual ③Feel ④Always feel |
| H12 | Do you feel pleasant in general?  ①Pleasant ②As usual ③Unpleasant ④Very unpleasant |
| H13 | Do you particularly like to play with boys when you were child?  ①I like it very much ②I like it ③JUST so ④I don't like it ⑤I don't like it very much |
| H14 | Do you agree with your own sexual orientation?  ① Strongly agree ②Somewhat agree ③Generally (indifferent) ④Disagree ⑤Strongly disagree |
| H15 | Do you tend to keep the privacy of your homosexual love?  ①Yes ②No ③It doesn't matter |
| H16 | Have you made your sexual orientation and identity public?  ①Never ②Open to family ③Open to important friends  ④Open only around a small range ⑤Completely open |
| H17 | Do you want to be recognized for your sexual orientation and identity?  ①Very desire ②desire ③general ④ a little desire ⑤don't care |
| H18 | Do you think the people around you think differently about you?  ①Always ② Often ③Generally ④Occasionally ⑤No |
| H19 | Does internet homosexual culture help you determine your sexual orientation?  ①Very helpful ②More helpful ③Generally ④A little helpful ⑤Not at all |

**Thank you again for participating in this survey!**

Investigate member:(signature) The reviewer:(signature)

Survey date:

**Supplementary Information**

**Additional file 1.** Questionnaire on psychological health status among transgender women in Shandong province, China. The questionnaire collected information about socio-demographic characteristics, HIV/AIDS cognition, related sexual behaviors, substance abuse, social support, gender identity and other related factors. In addition, supplementary figure 1 showed the correct rate of AIDS related knowledge.
